# Inflammasome-dependent IL-1*β* release depends upon membrane permeabilisation

**DOI:** 10.1038/cdd.2015.176

**Published:** 2016-02-12

**Authors:** F Martín-Sánchez, C Diamond, M Zeitler, A I Gomez, A Baroja-Mazo, J Bagnall, D Spiller, M White, M J D Daniels, A Mortellaro, M Peñalver, P Paszek, J P Steringer, W Nickel, D Brough, P Pelegrín

**Affiliations:** 1 Grupo de Inflamación Molecular, Centro de Investigación Biomédica en Red en el Área Temática de Enfermedades Hepáticas y Digestivas, Hospital Clínico Universitario Virgen de la Arrixaca, Instituto Murciano de Investigación Biosanitaria (IMIB-Arrixaca), Murcia, Spain; 2 Faculty of Life Sciences, University of Manchester, Manchester, UK; 3 Singapore Immunology Network (SIgN), Agency for Science Technology and Research (A*STAR), Singapore; 4 Heidelberg University Biochemistry Center, Heidelberg, Germany; 5 Probelte Biotechnology, S.L., Murcia, Spain

## Abstract

Interleukin-1*β* (IL-1*β*) is a critical regulator of the inflammatory response. IL-1*β* is not secreted through the conventional ER–Golgi route of protein secretion, and to date its mechanism of release has been unknown. Crucially, its secretion depends upon the processing of a precursor form following the activation of the multimolecular inflammasome complex. Using a novel and reversible pharmacological inhibitor of the IL-1*β* release process, in combination with biochemical, biophysical, and real-time single-cell confocal microscopy with macrophage cells expressing Venus-labelled IL-1*β*, we have discovered that the secretion of IL-1*β* after inflammasome activation requires membrane permeabilisation, and occurs in parallel with the death of the secreting cell. Thus, in macrophages the release of IL-1*β* in response to inflammasome activation appears to be a secretory process independent of nonspecific leakage of proteins during cell death. The mechanism of membrane permeabilisation leading to IL-1*β* release is distinct from the unconventional secretory mechanism employed by its structural homologues fibroblast growth factor 2 (FGF2) or IL-1*α*, a process that involves the formation of membrane pores but does not result in cell death. These discoveries reveal key processes at the initiation of an inflammatory response and deliver new insights into the mechanisms of protein release.

Interleukin-1*β* (IL-1*β*) is a proinflammatory cytokine that is central for host responses to infection.^1^ IL-1*β* is produced as an inactive precursor called pro-IL-1*β* mainly by inflammatory cells of myeloid lineage. Pro-IL-1*β* is rapidly induced upon exposure of inflammatory cells to pathogen-associated molecular patterns (PAMPs) or damage-associated molecular patterns (DAMPs) that bind to pattern recognition receptors (PRRs) to upregulate proinflammatory gene expression.^2^ A cell expressing pro-IL-1*β* is suggested to be ‘primed' and in order for the secretion of active mature IL-1*β* molecules the primed cell must encounter an additional PAMP or DAMP stimulation (signal 2) to activate cytosolic PRRs, often of the NLR (Nucleotide-binding domain and Leucine-rich repeat containing Receptor) family, to form large multiprotein complexes called inflammasomes.^3^ The best characterised inflammasome is formed by the PRR NLRP3 (NACHT, LRR, and PYD domains containing protein 3).^4^ Inflammasomes are composed of the PRR, pro-caspase-1, and an adaptor protein called ASC (apoptosis-associated speck-like protein containing a caspase recruitment domain) that interact via homology binding domains.^3^ Following activation, inflammasomes cause the activation of caspase-1 and the processing of pro-IL-1*β* to a mature form that is secreted.

IL-1*β* does not traffic through the ER or Golgi and the precise mechanisms of its secretion are poorly defined. Following a recent review of the literature, we have postulated that there are a number of possible mechanisms through which IL-1*β* could exit the cell.^5^ Potential secretory mechanisms include its release through the regulated secretion of lysosomes,^6^ the shedding of microvesicles from the plasma membrane,^7^ or its direct release across a hyperpermeable plasma membrane.^8^ This last mechanism is perhaps related to cell death that in many instances is linked to IL-1*β* secretion. Interestingly, another nonconventionally secreted protein, fibroblast growth factor 2 (FGF2), is secreted by membrane insertion of FGF2 oligomers as intermediates in FGF2 membrane translocation as part of its secretory mechanism.^9^ Despite having very little sequence homology, FGF2 and IL-1*β* are structural homologues with both containing a *β*-barrel structure.^10, 11^ Thus, it is possible that mature IL-1*β* could create membrane translocation pores in a manner analogous to FGF2 to facilitate its release.

Using the canonical inducer of NLRP3 inflammasome activation, extracellular ATP (acting via the P2X7 receptor), we previously reported that release of IL-1*β* preceded that of the cell death marker lactate dehydrogenase (LDH) from primary peritoneal macrophages.^12^ However, IL-1*β* was secreted just before LDH was released, suggesting that the cells were already committed to cell death by the time they secreted IL-1*β*.^12^ However, it seems unlikely that IL-1*β* release can only be linked to cell death and IL-1*β* is released from human monocytes or neutrophils in the absence of cell death,^13, 14^ suggesting that there are perhaps cell-specific mechanisms of release. Here we used sensitive single-cell quantitative approaches in addition to new pharmacological interventions with membrane stabilising reagents to show that in response to NLRP3 inflammasome activation, the secretion of IL-1*β* from macrophages depended upon permeabilisation of the plasma membrane, occurred concomitantly with cell death, and was distinct from the specific formation of translocation pores utilised by FGF2.

## Results

### IL-1*β* does not possess FGF-2-like properties of membrane pore formation

**Figure 1:**
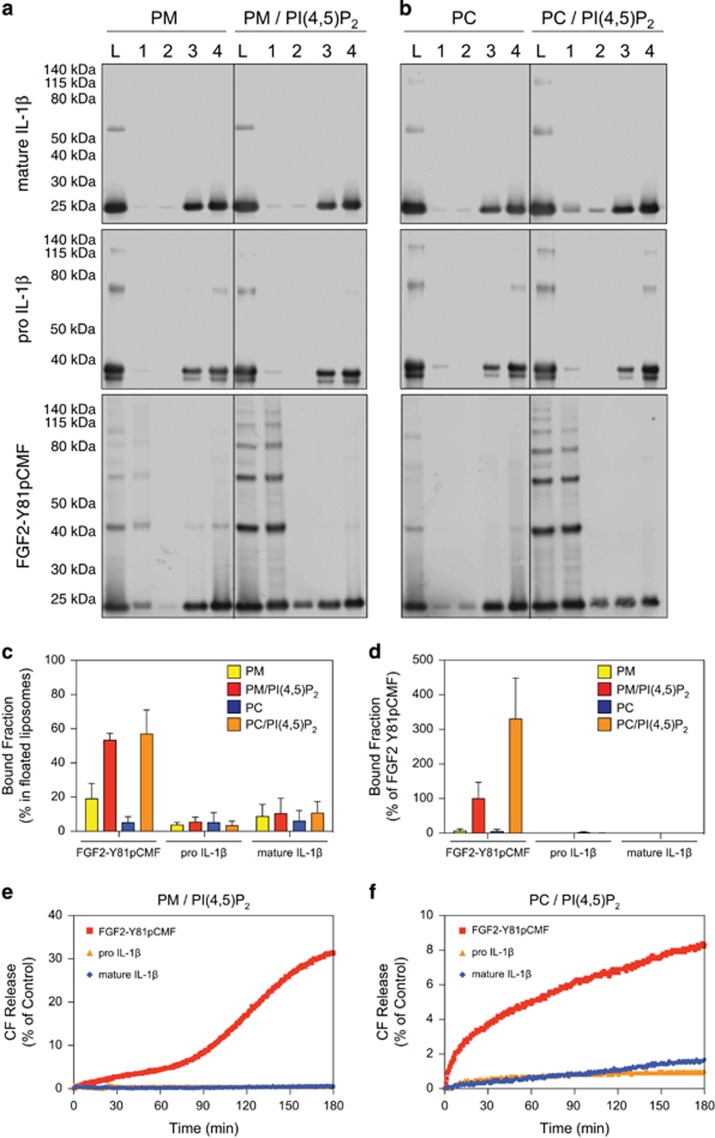
Membrane binding and pore formation properties of pro-IL-1*β*, mature IL-1*β*, and phosphomimetic FGF2. (**a**–**c**) Biochemical analysis using liposomes characterised by either a plasma membrane-like lipid composition (PM) with or without 2 mol% PI(4,5)P_2_ (panel A) or phosphatidylcholine (PC) liposomes with or without 10 mol% PI(4,5)P_2_ (**b**). Binding properties of mature IL-1*β*, pro-IL-1*β*, and FGF2-Y81pCMF were compared. Membrane-bound material (fraction 1) was separated from free proteins (fractions 2–4) by membrane flotation in density gradients. Fractions were analysed by SDS-PAGE and western blotting using anti-IL-1*β* and anti-FGF2 antibodies, respectively. Quantification was done using an Odyssey infrared imaging system (LI-COR Bioscience). The amount of protein found in fraction 1 was quantified as the percentage of the total signal from fractions 1–4 (**c**). Mean values with S.D. of three independent experiments (*n*=3) are shown. (**d**) Binding of GFP-tagged variants of mature IL-1*β*, pro-IL-1*β*, and FGF2-Y81pCMF to liposomes with various lipid compositions as indicated was examined using an assay based upon flow cytometry. Signals were normalised based on FGF2-Y81pCMF binding to PM liposomes containing PI(4,5)P_2_ that was set to 100%. Mean values with S.D. (*n*=3) are shown. (**e** and **f**) Liposomes containing luminal carboxyfluorescein either consisting of a plasma membrane-like lipid composition (PM) containing PI(4,5)P_2_ (**e**) or phosphatidylcholine (PC) containing PI(4,5)P_2_ (**f**) were used to monitor membrane integrity upon incubation with the proteins indicated. Membrane pore formation was detected by the release of carboxyfluorescein that can be measured through dequenching. The results shown are representative for three independent experiments

FGF2 secretion is a specific and tightly regulated process that involves direct translocation across plasma membranes in a process that involves PI(4,5)P_2_-dependent membrane recruitment,^15^ oligomerisation,^9^ and membrane pore formation,^9, 16^ and is regulated by tyrosine phosphorylation of FGF2 mediated by Tec kinase.^17^ Given the structural similarity between FGF2 and IL-1*β*, and that the secretion of IL-1*β* has recently been reported to potentially involve changes in plasma membrane integrity,^8^ we hypothesised that IL-1*β* may also be capable of binding to PI(4,5)P_2_ concomitant with the formation of membrane pores. Both authentic and fluorescently labelled variants of phosphomimetic FGF2,^9^ pro-IL-1*β*, and IL-1*β* were expressed and purified to homogeneity. In a first set of experiments, authentic variants were tested with regard to binding to PI(4,5)P_2_-containing liposomes using classical biochemical flotation experiments (Figures 1a and b). Fraction 1 contains protein that bound to membranes, and fraction L is the load of the gradient. Although phosphomimetic FGF2 was found to bind efficiently to PI(4,5)P_2_-containing liposomes, neither pro-IL-1*β* nor mature IL-1*β* bound to PI(4,5)P_2_ to a significant extent (Figures 1a–c). Consistent with previous studies,^9, 16^ these experiments revealed the formation of SDS-resistant FGF2 oligomers that form upon membrane binding. These findings could be confirmed with fluorescently labelled forms of phosphomimetic FGF2, pro-IL-1*β*, and IL-1*β* using a flow cytometry setup to quantify protein–lipid interactions.^18^ Similar to the flotation experiments (Figures 1a–c), neither pro-IL-1*β* nor mature IL-1*β* bound to membranes, whereas phosphomimetic FGF2 showed strong membrane binding that was dependent on the presence of PI(4,5)P_2_ (Figure 1d). In another set of experiments, we tested whether pro-IL-1*β* or mature IL-1*β* could permeabilise membranes *in vitro* analysing membrane passage of a small fluorescent tracer.^9, 19^ As shown in Figures 1e and f, a membrane permeabilising activity of these proteins could not be observed. In contrast, as reported previously, phosphomimetic FGF2 forms membrane pores in a PI(4,5)P_2_-dependent manner resulting in membrane passage of the fluorescent tracer. These data demonstrate that neither pro-IL-1*β* nor mature IL-1*β* has properties resembling those of FGF2 regarding membrane binding and pore formation.

### IL-1*β* release depends upon plasma membrane permeabilisation

**Figure 2:**
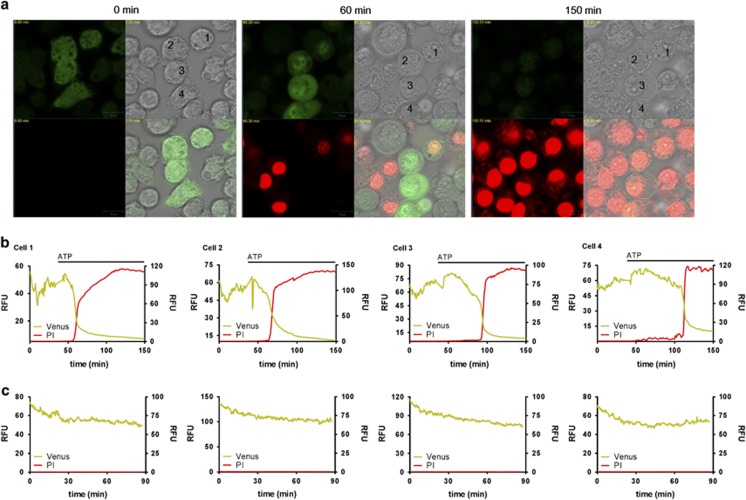
Real-time secretion of IL-1*β*. Immortalised mouse BMDMs expressing IL-1*β*Venus were treated with LPS (1 *μ*g/ml, 4 h). Fluorescence of IL-1*β*Venus (green) and PI (red) was then observed when treated with or without ATP (5 mM). (**a**) Shown are images of brightfield (upper right quad), Venus (upper left quad), PI (lower left quad), and merged (lower right quad). Shown are images for time 0, 60, and 150 min. Raw fluorescence data from cells labelled 1–4 are shown in (**b**) The fluorescence traces shown in (**c**) are from control movies where no ATP was added. Scale bar represents 10 *μ*m

To further interrogate the mechanisms of IL-1*β* release from macrophages, we transformed immortalised mouse bone marrow-derived macrophages (BMDMs) with a lentiviral vector to express pro-IL-1*β* as a C-terminal fluorescent Venus protein fusion. In these cells the distribution of pro-IL-1*β*Venus was cytosolic, as previously described for endogenous pro-IL-1*β*,^12^ and was processed and released in response to lipopolysaccharide (LPS) and ATP stimulation (Supplementary Figure 1). Furthermore, the temporal pattern of IL-1*β* release and membrane permeabilisation/cell death (as assessed by the release of the soluble cytosolic protein LDH that leaks from the cell during necrosis) were similar between pro-IL-1*β*Venus-transduced cells and wild-type (WT) cells (Supplementary Figure 1). Consistent with the requirement of priming for NLRP3 expression,^20^ IL-1*β* was not released without prior LPS stimulation (Supplementary Figure 1). Using these cells we used time-lapse confocal microscopy to observe the secretion of IL-1*β* (Supplementary Movies 1 and 2). LPS-primed cells were incubated in the presence or absence of ATP and Venus fluorescence was continuously monitored. To simultaneously monitor membrane integrity we included the cell-impermeant nucleic acid stain propidium iodide (PI) in the cell culture medium. Following the addition of ATP there was a drop in Venus fluorescence that correlated with an increase in PI fluorescence, suggesting that the release of IL-1*β* coincided with membrane permeabilisation (Figures 2a and b). Shown are the raw data from four cells identified in the movie snapshots (Figure 2b). In control cells, where no ATP was added, there was some reduction of the Venus fluorescent signal and no increase in PI fluorescence (Figure 2c and Supplementary Movie 3). These data suggest that IL-1*β* secretion from macrophages occurs across a hyperpermeable plasma membrane.

**Figure 3:**
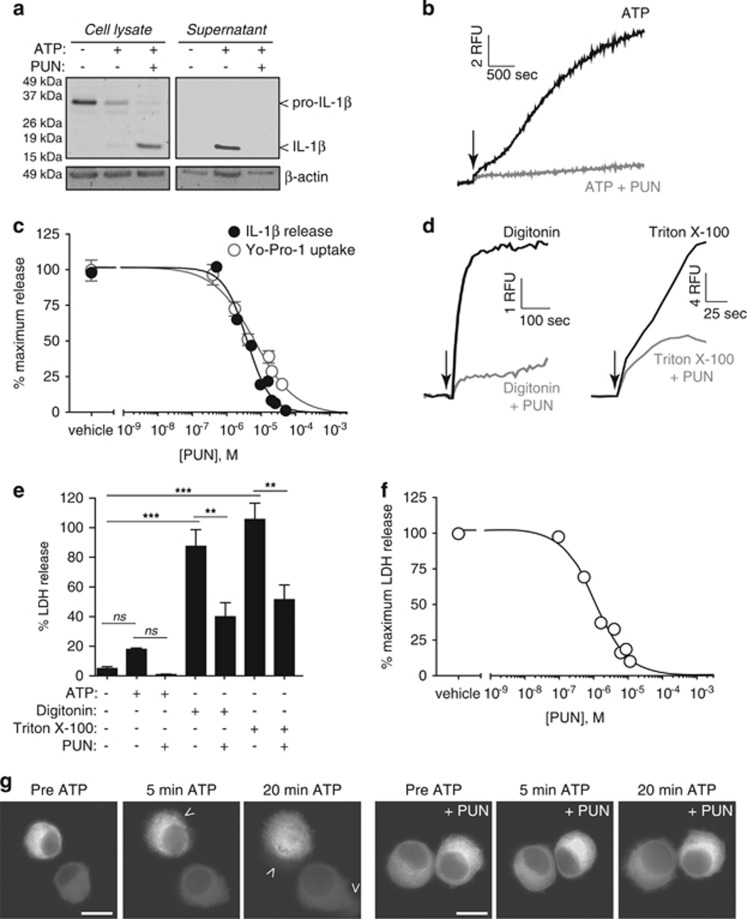
Punicalagin blocks mature IL-1*β* release and membrane permeabilisation. (**a**) Immunoblot analysis of the processing and release of pro-IL-1*β* in cell lysates and supernatants of LPS-primed (1 *μ*g/ml, 4 h) BMDM unstimulated (−) or stimulated (+) for 30 min with ATP (5 mM) in the presence (+) or absence (−) of punicalagin (PUN; 25 *μ*M). (**b**) Kinetics of Yo-Pro uptake in BMDMs treated as in (**a**). (**c**) Punicalagin concentration–inhibition curves obtained for IL-1*β* release and Yo-Pro uptake in BMDMs treated as in (**a**); punicalagin IC_50_ values were 3.91 and 7.65 *μ*M for IL-1*β* release and Yo-Pro uptake respectively. (**d**) Kinetics of Yo-Pro uptake in LPS-primed BMDMs treated with digitonin (50 *μ*M) or Triton X-100 (0.1%) in the presence or absence of punicalagin (PUN; 25 *μ*M). (**e**) Percentage of extracellular LDH from BMDM treated as in (**a**) or for 30 min with detergents as in (**d**) in the presence (+) or absence (−) of punicalagin (PUN; 25 *μ*M). **P<0.005; ***P<0.001; n.s., not significant (*P*>0.05) difference (ANOVA with Bonferroni's post-test). (**f**) Punicalagin concentration–inhibition curves obtained for LDH release in BMDMs treated as in (**a**); punicalagin IC_50_ was 3.67 *μ*M. (**g**) Deconvolved images of immortalised macrophages expressing pro-IL-1*β*Venus and stimulated as in (**a**). Shown are images of Venus fluorescence for time 0 (pre-ATP) and 5 or 20 min after ATP application; see Supplementary Movies 4 and 5. Scale bar represents 10 *μ*m; arrowheads indicate areas where the fluorescence is leaking from the cell

The mechanism of IL-1*β* release was then further interrogated using the complex polyphenolic compound punicalagin (Supplementary Figure 2A). We discovered that punicalagin prevented ATP-induced IL-1*β* secretion, but not its processing. Therefore, after ATP treatment in the presence of punicalagin, mature IL-1*β* was associated with macrophage cell lysates (Figure 3a). Punicalagin has a complex molecular structure, and the full intact molecular structure was required to inhibit IL-1*β* release, as its component parts (punicalin, ellagic acid, and urolithin) had no effect on IL-1*β* secretion (Supplementary Figures 2A and B). Pomegranate extract (Pomanox, Probelte Biotechnology S.L., Murcia, Spain) with a punicalagin content of 20% was also effective at preventing mature IL-1*β* release from ATP-treated macrophages (Supplementary Figure 2C). In addition, punicalagin was a potent blocker of plasma membrane permeabilisation (Yo-Pro-1 uptake) in response to ATP (Figure 3b), and the half-maximal inhibitory concentration (IC_50_) for IL-1*β* release and membrane permeabilisation were 3.91 and 7.65 *μ*M, respectively (Figure 3c). Punicalagin-mediated preservation of plasma membrane integrity was also evident measuring Yo-Pro-1 uptake after cell treatment with detergents (Figure 3d) and measuring the release of LDH to the cell supernatant following ATP treatment or detergent application (Figure 3e). Punicalagin was able to maintain the integrity of macrophages treated with detergents (Supplementary Figure 3A), although this protection was overcome as the dose of detergent applied was increased (Supplementary Figure 3B). As further controls, punicalagin did not quench Yo-Pro-1 fluorescence, nor did it directly inhibit LDH enzyme activity (Supplementary Figures 3C and D). The IC_50_ of punicalagin against ATP-induced LDH release was 3.67 *μ*M, and therefore very similar to the IC_50_ for blocking IL-1*β* release (Figure 3f). Punicalagin treatment of immortalised macrophages expressing pro-IL-1*β*Venus was able to maintain cell integrity and retain fluorescence leaking from the cell after ATP application (Figure 3g and supplementary Figure 3E and Supplementary Movies 4 and 5).

### Loss of membrane integrity is not required for inflammasome formation

**Figure 4:**
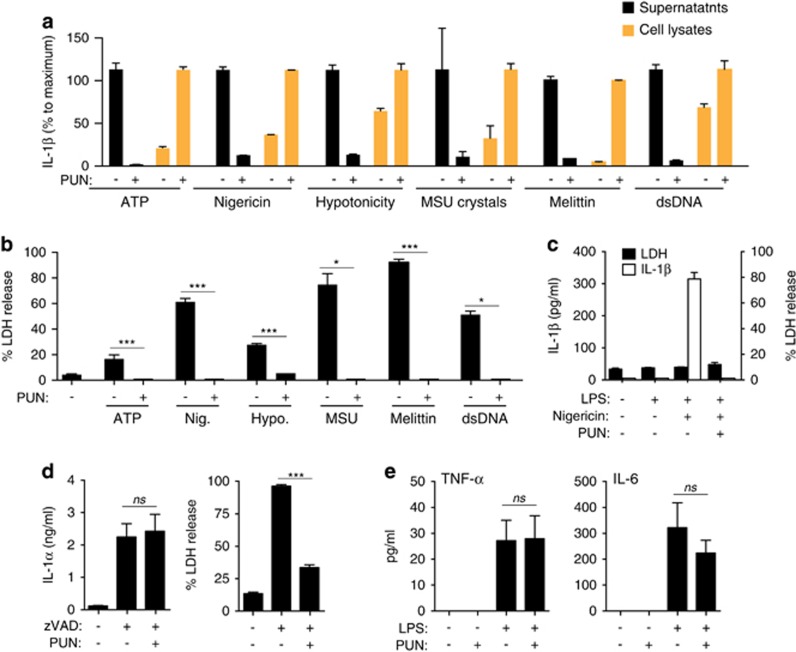
Punicalagin retain mature IL-1*β* in response to different NLRP3 activators. (**a**) IL-1*β* ELISA in cell lysates (orange bars) or supernatants (black bars) from BMDMs primed with LPS (1 *μ*g/ml, 4 h) followed by no stimulation (−) or stimulation with ATP (5 mM, 30 min), nigericin (10 *μ*m, 30 min), hypotonic solution (90 mOsm, 1 h), monosodium urate crystal (MSU; 200 *μ*g/ml, 3 h), melittin (5 *μ*M, 30 min), or double-stranded DNA (dsDNA; 2 *μ*g/ml, 30 min) in the absence or presence of punicalagin (PUN; 25 *μ*M). (**b**) Extracellular LDH from macrophages treated as in (**a**). (**c**) IL-1*β* ELISA in supernatants (white bars) or extracellular LDH (black bars) from mouse bone marrow-isolated neutrophils primed with LPS (100 ng/ml, 4 h) followed by no stimulation (−) or stimulation with nigericin (10 *μ*m, 30 min) in the absence or presence of punicalagin (PUN; 25 *μ*M). (**d**) IL-1*α* ELISA in supernatants (left panel) or extracellular LDH (right panel) from BMDMs primed with LPS (1 *μ*g/ml, 4 h) followed by no stimulation (−) or stimulation (+) with zVAD (100 *μ*M, 20 h) in the absence or presence of punicalagin (PUN; 25 *μ*M). (**e**) TNF-*α* and IL-6 ELISA in supernatants from BMDMs primed with LPS (1 *μ*g/ml, 4 h), washed, and followed by incubation for 30 min in the absence or presence of punicalagin (PUN; 25 *μ*M). *P<0.05; ***P<0.001 (Student's *t*-test)

We then studied IL-1*β* secretion in response to different NLRP3 inducers and also in response to AIM2 inflammasome activation. We used an ELISA with higher sensitivity for mature IL-1*β* than for pro-IL-1*β*, and validated it comparing western blot data from LPS-primed macrophage cell lysates treated with or without ATP and punicalagin (Supplementary Figure 4A). This ELISA detected more IL-1*β* in lysates with mature IL-1*β* than in lysates with just pro-IL-1*β*. Using this ELISA approach, we found that punicalagin blocked IL-1*β* release in response to different NLRP3 inflammasome and AIM2 activators, and mature IL-1*β* was retained in the cell lysate after activation of macrophages with nigericin, hypotonicity, uric acid crystals, the pore-forming toxin melittin, and dsDNA (Figure 4a). Similarly, punicalagin significantly reduced release of LDH in response to different NLRP3 and AIM2 inflammasome activators (Figure 4b). Punicalagin did not affect the cellular volume changes of macrophages in response to hypotonicity (Supplementary Figure 4B), suggesting that it was not acting as an external osmolyte to resist osmotic swelling and cell lysis. Therefore, a change in plasma membrane permeability emerges as a common mechanism for the unconventional release of IL-1*β* from macrophages. Punicalagin also inhibited the release of IL-1*β* from neutrophils, a cell type that releases IL-1*β* independently of cell death (Figure 4c). Furthermore, punicalagin inhibited necroptotic cell death but not the unconventional release of IL-1*α* that we previously reported in response to necroptotic stimuli^21^ (Figure 4d). The specificity of the effect of punicalagin to IL-1*β* release was further demonstrated by its failure to inhibit the conventional release of TNF-*α* and IL-6 (Figure 4e).

**Figure 5:**
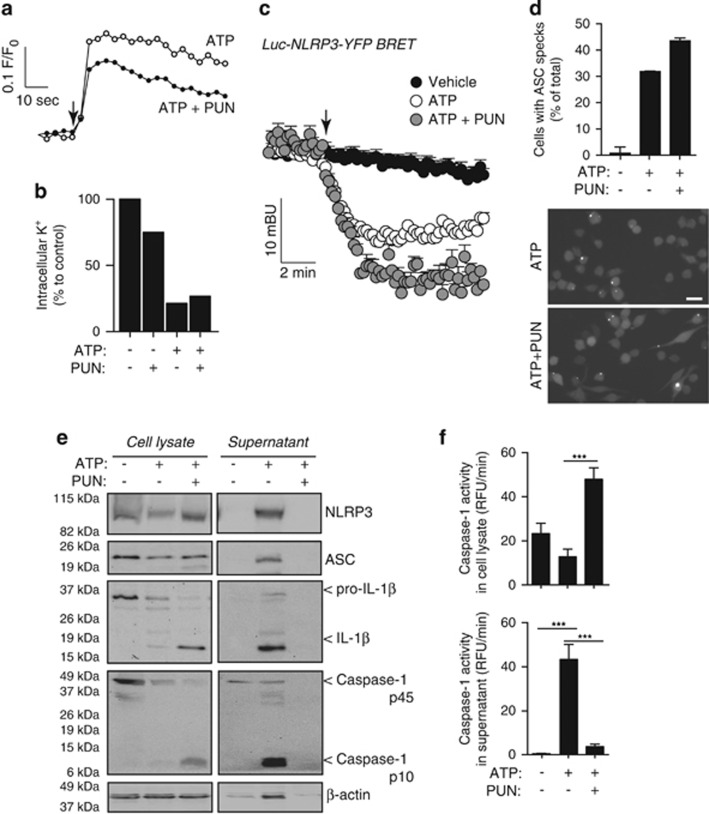
Punicalagin does not block NLRP3 or caspase-1 activation. (**a**) Intracellular Ca^2+^ rise in mouse BMDMs primed with LPS (1 *μ*g/ml, 4 h) followed by stimulation with ATP (1 mM, added when indicated with an arrow) in the absence or presence of punicalagin (PUN; 25 *μ*M). (**b**) Relative intracellular K^+^ concentration of BMDMs treated as in (**a**) with ATP for 30 min. (**c**) Kinetic of net BRET signal for NLRP3 protein expressed in P2X7-HEK293 cells unstimulated or stimulated with ATP (5 mM, added when indicated with an arrow). (**d**) Average quantification (top) and fluorescence microscopy images (bottom) of immortalised ASC-Cherry macrophages containing ASC specks treated as in (**b**); *n* >400 cells/condition from 2 independent experiments; scale bar represents 20 *μ*m. (**e** and **f**) Immunoblot analysis (**e**) and caspase-1 activity measurements (**f**) of cell lysate and supernatant of BMDMs treated as in (**b**); ***P<0.001 difference (Student's *t*-test)

Punicalagin did not directly block P2X7 receptor activation, as assayed by increase of cytosolic Ca^2+^ or decrease of intracellular K^+^ (Figures 5a and b), cations that directly permeate through the P2X7 receptor ion channel.^22^ Punicalagin did not directly interfere with NLRP3 activation as measured by BRET (Figure 5c), nor did it impair the formation of ASC specks (Figure 5d). In addition, punicalagin did not inhibit caspase-1 activity after NLRP3 inflammasome activation (Figures 5e and f), but it completely prevented release of active caspase-1 (p10 subunit), ASC, and NLRP3 inflammasome components (Figure 5e).

To investigate the role of the inflammasome on plasma membrane destabilisation and cell death, we used macrophages deficient in NLRP3, ASC, or caspase-1 primed with LPS and then treated with ATP. These macrophages also presented membrane permeabilisation when compared with WT macrophages, but LDH release was abolished (Supplementary Figures 4C and D). Punicalagin was able to block plasma membrane permeabilisation in both WT and inflammasome-deficient macrophages (Supplementary Figures 4C and D).

### Pharmacological and kinetic characterisation of IL-1*β* release

**Figure 6:**
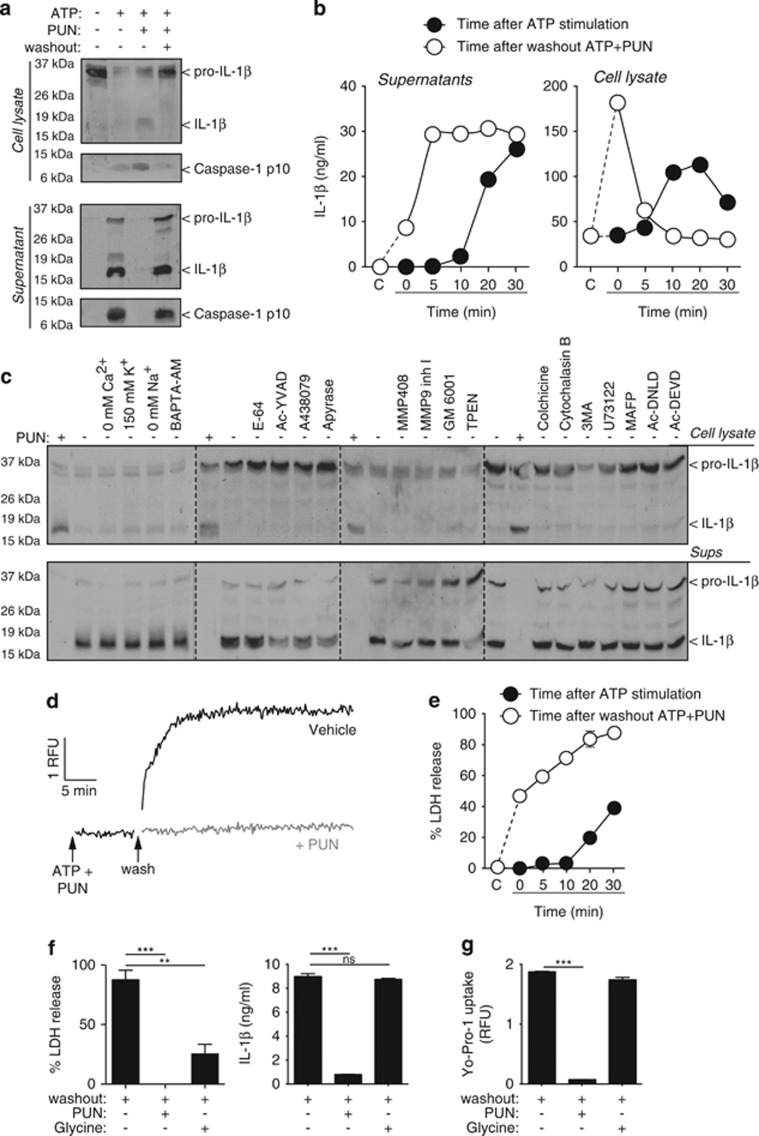
IL-1*β* release pharmacology. (**a**) Immunoblot analysis of cell lysate and supernatant of mouse BMDMs primed with LPS (1 *μ*g/ml, 4 h), followed by no stimulation (−) or stimulation (+) with ATP (5 mM, 20 min) in the absence (−) or presence (+) of punicalagin (PUN; 25 *μ*M) and then washout (+) or not (−) for 20 min. (**b**) ELISA of IL-1*β* of cell lysate and supernatant from BMDMs primed as in (**a**). Measures are taken every 5 min during 30 min of ATP stimulation (5 mM) after priming (black circles) or washout after 30 min stimulation with ATP (5 mM) with punicalagin (PUN; 25 *μ*M) (white circles). (**c**) Immunoblot analysis of cell lysate and supernatant of BMDMs treated as in (**a**) and during washout after ATP+PUN cells were incubated with punicalagin (PUN; 25 *μ*M), in a buffer without Ca^2+^, high K^+^ (150 mM), with NMDG^+^ (0 mM Na^+^), or normal ion buffer with BAPTA-AM (100 *μ*M), E-64 cathepsin inhibitor (50 *μ*M), Ac-YVAD caspase 1 inhibitor (100 *μ*M), A438079 P2X7 antagonist (25 *μ*M), apyrase (3 U/ml), MMP408 (1 *μ*M), MMP9 (0.5 *μ*M) and GM6001 (0.5 *μ*M) metalloprotease inhibitors, TPEN Zn^2+^ chelator (50 *μ*M), colchicine (50 *μ*M) and cytochalasin B (2.5 *μ*g/ml), 3-MA autophagy inhibitor (6 mM), U73122 phospholipase C inhibitor (10 *μ*M), MAFP phospholipase A inhibitor (10 *μ*M), Ac-DNLD, or Ac-DEVD caspase-3 inhibitors (100 *μ*M). (**d** and **e**) Kinetic of Yo-Pro uptake (**d**) and percentage of extracellular LDH (**e**) from macrophages treated as in (**b**). (**f**) Percentage of extracellular LDH release and ELISA of IL-1*β* in supernatant from BMDMs treated as in (**a**) and during washout after ATP+PUN cells were incubated with punicalagin (PUN; 25 *μ*M) or glycine (5 mM). (**g**) Yo-Pro uptake in BMDMs treated as in (**f**). **P<0.01; ***P<0.001; n.s., not significant (*P*>0.05) difference (ANOVA with Bonferroni multiple comparison test)

After washing punicalagin from macrophages treated with ATP, IL-1*β* and the p10 fragment of caspase-1 were released without the requirement of additional ATP stimulation (Figure 6a). To date, the study of IL-1*β* release has been limited to the period of time immediately following inflammasome activation and IL-1*β* processing. However, using punicalagin, we were now able to study IL-1*β* secretion independently of inflammasome activation. IL-1*β* release occurred within the first 5 min after the removal of punicalagin (Figure 6b). This release was paralleled by a decrease of intracellular IL-1*β* (Figure 6b). Contrary to NLRP3 inflammasome activation, the release mechanism of IL-1*β* was independent of K^+^ efflux or changes in intracellular Ca^2+^ or Na^+^ (Figure 6c). The IL-1*β* release mechanism was also independent of the P2X7 receptor or purinergic signalling, cathepsin, or caspase-1 activity (Figure 6c). Metalloprotease inhibition or Zn^2+^ chelation also failed to block IL-1*β* release (Figure 6c). The activity of apoptotic caspase-3 and -7, phospholipases A and C, autophagy, or tubulin and actin cytoskeleton dynamics were also not required for the release of IL-1*β* (Figure 6c). In addition, after washing punicalagin there was a fast membrane permeabilisation and release of LDH (Figures 6d and e), suggesting that IL-1*β* release was dependent upon membrane permeabilisation. However, LDH release was mechanistically overlapping with the increase of plasma membrane permeability involved in the release of IL-1*β*, as treatment of the cells with the cytoprotective agent glycine after washing punicalagin prevented the release of LDH and the p10 active fragment of caspase-1 from the cell, without affecting the release of mature IL-1*β* or permeabilisation of the plasma membrane (Figures 6f and g and Supplementary Figure 4E).

**Figure 7:**
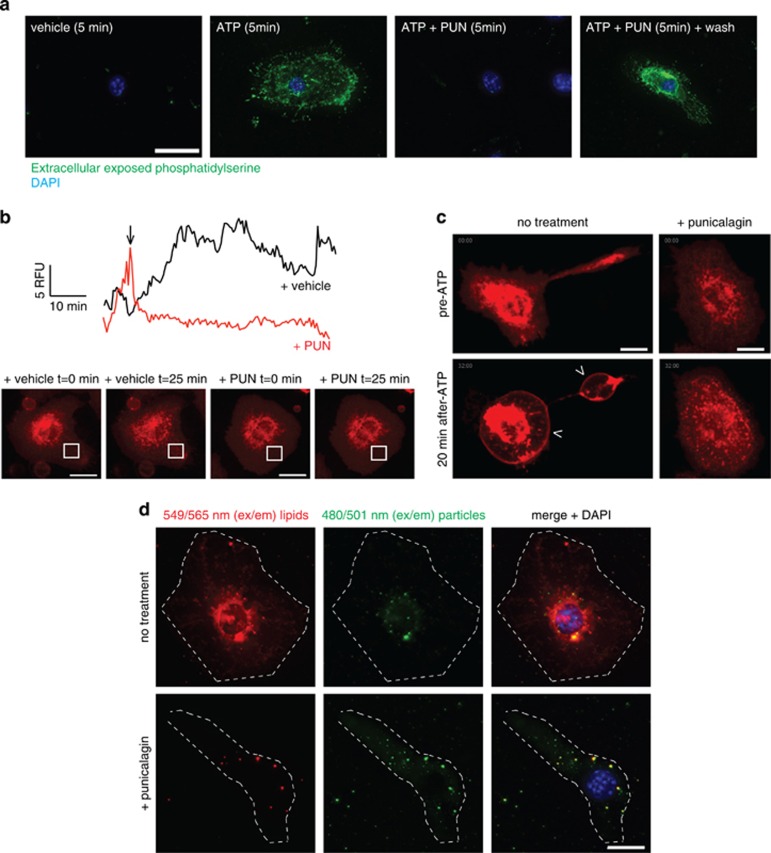
Punicalagin stabilises plasma membrane lipids. (**a**) Deconvolved maximum projection fluorescence images of representative BMDMs primed with LPS (1 *μ*g/ml, 4 h), followed by no stimulation (vehicle) or stimulation with ATP (3 mM) for 5 min in the absence or presence of punicalagin (PUN, 25 *μ*m) and then labelled with FITC-annexin V. Nuclei stained with DAPI (blue); scale bar represents 20 *μ*m. (**b**) Mean of relative fluorescence unit (RFU) quantification in different regions of interest of the plasma membrane, as indicated in the images inserted, of macrophages primed with LPS as in (**a**) and then labelled with cholera toxin B-Alexa fluor 647 (CTB), untreated (vehicle, black trace), or treated with punicalagin (PUN, 25 *μ*m, red trace) from the time indicated with an arrow. Movements of stained cholesterol-rich patches with CTB results in variations of RFU on the selected ROI, and punicalagin prevented these movements; see Supplementary Movies 6 and 7. (**c**) Deconvolved images of representative BMDMs stimulated as in (**a**), but with 5 mM of ATP and stained with CTB; shown are images of CTB fluorescence for time 0 (pre-ATP) and 20 min after ATP application; see Supplementary Movies 8 and 9. Scale bar represents 10 *μ*m. (**d**) Deconvolved maximum projection fluorescence images of representative BMDMs primed with LPS as in (**a**) and incubated for 2 h with the Fuse-It liposomes in the absence or presence of punicalagin. Cells are visualised with red-labelled membranes, green-labelled cell lumen, and nuclei counterstained with DAPI (blue). Scale bar represents 10 *μ*m, cellular edges are shown with a white dotted line

Punicalagin prevented phosphatidylserine flip in macrophages after ATP stimulation (Figure 7a), and after punicalagin washout phosphatidylserine was quickly exposed to the outer bilayer (Figure 7a), suggesting its effects were reversible. To further study the effect of punicalagin on plasma membrane fluidity/stability, we labelled cholesterol-rich rafts with cholera toxin B-AlexaFluor 647 and followed the dynamics of the rafts over time by quantifying the mean fluorescence intensity in different regions of interest of the plasma membrane. Addition of punicalagin dramatically attenuated the movement of rafts on the plasma membrane (Figure 7b and Supplementary Movies 6 and 7) and also prevented bleb formation in response to ATP (Figure 7c and Supplementary Movies 8 and 9). Furthermore, punicalagin was able to impair lipid distribution among plasma membrane using liposomes consisting of fluorescently 549/565 nm (em/ex)-labelled amphipathic molecules (Figure 7d), but it did not impair delivery of fluorescently 480/501 nm (em/ex)-labelled marker particles contained within the lumen of liposomes into the cell cytosol (Figure 7d). Therefore, punicalagin stabilises lipids on the plasma membrane after ATP stimulation, and this lipid stabilisation is important for IL-1*β* release.

## Discussion

The vast majority of proteins that are secreted from the cell depend upon a signal peptide at their N-terminus that directs their trafficking into the ER, through which they transit to the Golgi and are then exocytosed from the cell.^23^ However, there are a small number of important proteins that lack a signal peptide and do not utilise the ER–Golgi route of protein secretion.^24, 25^ The secretion of the proinflammatory cytokine IL-1*β* is an enduring question spanning almost 30 years, since the discovery that IL-1*β* lacks a signal peptide^19^ and does not traffic through the ER and Golgi.^26^ Despite a number of interesting ideas, all supported by biochemical data, no consensus for a mechanism of IL-1*β* secretion exists.^5^ However, given its contribution to major disease states,^27^ understanding the mechanisms of IL-1*β* secretion may allow us to identify new therapeutic targets for the treatment of inflammatory disease.

Despite sharing little sequence homology, IL-1*β* and the unconventionally secreted protein FGF2 are close structural homologues.^10, 11^ One of the proposed mechanisms for the secretion of IL-1*β* has been its direct release across the plasma membrane via a mechanism that is closely followed by lysis of the secreting cell.^12, 28^ Recent evidence using a live single-cell assay to measure released IL-1*β* suggests that changes in membrane permeability are required for its secretion.^8^ Recent work has identified that FGF2 is directly secreted across the plasma membrane via a mechanism dependent upon its recruitment by the plasma membrane lipid PI(4,5)P_2_,^9, 16^ and thus it was tempting to speculate that these unconventionally secreted proteins may share a common mechanism of release. However, under the experimental conditions used here it would appear that the mechanisms of IL-1*β* and FGF2 secretion are distinct, with only FGF2 being able to directly permeabilise membranes in the assays used here. This is not to say, however, that the conditions of these assays did not favour the permeabilising effects of IL-1*β*, and that different experimental conditions are required. Conventional biochemical methods have limited the investigation into the processes involved in IL-1*β* secretion to population dynamics, although recent advances in single-cell assays have provided new insights into the dynamics of IL-1*β* secretion.^8^ To further examine the mechanisms of IL-1*β* secretion we developed a live single-cell real-time assay for measuring IL-1*β* release. Immortalised BMDMs transduced to express pro-IL-1*β* with a C-terminal Venus fluorescent protein tag allowed us to visualise IL-1*β* secretion using real-time single-cell confocal microscopy. Secretion of IL-1*β* from these cells after NLRP3 inflammasome activation with ATP appeared normal as assayed by biochemical techniques and the data on IL-1*β*Venus that we recorded were completely consistent with the study of Shirasaki *et al.*^8^ The secretion of IL-1*β*Venus correlated closely with the permeabilisation of the plasma membrane as measured by PI uptake. These data suggest that the secretion of IL-1*β* from macrophages following inflammasome activation is dependent upon permeabilisation of the plasma membrane.

We were able to confirm the requirement of plasma membrane permeabilisation using novel membrane stabilising agents that prevented the release of mature IL-1*β*, but that had no effect on inflammasome assembly and caspase-1 activation. Given the close relationship between IL-1*β* processing after inflammasome activation and its release,^12^ interventions that inhibit the release of IL-1*β* have done so exclusively by blocking inflammasome activation and thus inhibiting its processing to a mature form. We discovered that the complex polyphenolic compound punicalagin is a novel and potent specific inhibitor of the IL-1*β* release mechanism. These data are the first demonstration of an intervention blocking the release pathway of mature IL-1*β* in response to different NLRP3 activators. Previous studies on IL-1*β* release have focussed on NLRP3 activation via ATP application and P2X7 receptor signalling.^7, 8, 12, 29, 30^ Here we report that, in addition to ATP, punicalagin is also able to block mature IL-1*β* release from macrophages stimulated with nigericin, MSU crystals, or hypotonicity, the pore-forming toxin melittin and also in response to AIM2 activation via dsDNA, pointing to a common mechanism for the release of this cytokine. Entirely consistent with our data on the requirement of membrane permeabilisation, we identified that the mechanism of action for punicalagin appeared to be via plasma membrane lipid stabilisation and consequently inhibition of permeability. Punicalagin did not affect the release of IL-1*α*, or classically secreted cytokines, such as TNF-*α* or IL-6. Early events of cell death are characterised by phosphatidylserine exposure to the external plasma membrane bilayer,^31^ and P2X7 receptor activation results in a fast phosphatidylserine flip to the outer leaflet of the plasma membrane.^32^ Here we found that membrane stabilisation by punicalagin affected not only lipid fluidity, but also blocked P2X7 receptor-induced phophatidylserine flip and other markers of cell integrity (plasma membrane permeabilisation and LDH leakage), even when active caspase-1 was found intracellularly. Caspase-1 activity is suggested as the main driver of a specific programme of cell death termed pyroptosis that is associated with the leakage of intracellular proteins, including the release of large inflammasome complexes.^33, 34^ Our data show that both mechanisms, membrane permeability and caspase-1 activity, are important and independent mechanisms leading to IL-1*β* release from macrophages, with plasma membrane permeability dependent on signal 2 as it was unaffected in caspase-1-deficient macrophages. The specific membrane stabilising effects of punicalagin were rapidly reversible, and following its washout mature IL-1*β* and the inflammasome components NLRP3, ASC, and active caspase-1 were rapidly released. Punicalagin also stabilised the cell membrane to detergents and prevented LDH leaking in response to detergent or NLRP3 inflammasome activation, and furthermore it impaired necroptotic cell death, suggesting it has a broad effect on membrane stabilisation. Pharmacological characterisation of this release pointed to a process independent of caspase-1 activity, but occurred in parallel to the death of the macrophage. When using glycine as a cytoprotective agent,^35^ we found that after punicalagin washout, glycine was able to impair LDH and caspase-1 release, but did not prevent plasma membrane permeabilisation and IL-1*β* release. Together, these data allow us to propose a model of IL-1*β* release from macrophages after inflammasome activation that is a nonspecific loss of plasma membrane integrity. In cells such as neutrophils, where IL-1*β* release occurs in the absence of cell death,^14^ punicalagin also inhibited IL-1*β* release, indicating a common, although better controlled, secretion mechanism that in macrophages overlaps with cell death. Recently, the cytoplasmic protein gasdermin D (Gsdmd) was reported to be critical for membrane permeabilisation and IL-1*β* release during pyroptosis.^36, 37^ Gsdmd is a substrate for caspase-1 and -11 and cleavage allows the N-terminal fragment of Gsdmd to destabilise membranes, facilitating pyroptosis in both noncanonical and canonical NLRP3 pathways.^36, 37, 38^ The effects of punicalagin reported here are remarkably similar to the effects of knocking out Gsdmd, in that while release is inhibited, mature caspase-1 and IL-1*β* are retained inside the cell following NLRP3 activation.^37, 38^ Whether punicalagin is an inhibitor of Gsdmd cleavage, or of the N-terminal fragment's signalling pathway, or acts independently of Gsdmd, remains to be determined. However, the functional phenotype of Gsdmd knockout or punicalagin treatment is at present indistinguishable, and this report is the first to describe an intervention of this kind.

## Materials and Methods

### Reagents

LPS, DAPI (4,6-diamidino-2- phenylindol), ATP, Triton X-100, digitonin, apyrase, TPEN, colchicine, cytochalasin B, 3-methyladenine, U73122, methyl arachidonyl fluoophosphonate, ellagic acid, glycine, and nigericin were from Sigma (Madrid, Spain); Punicalagin ≥98% (HPLC) was also from Sigma, but in some experiments an aqueous pomegranate extract produced by Probelte Biotechnology S.L. (Pomanox) with a content in punicalagin of 20% (w/w) was used to compare efficacy of the extract with the HPLC-purified punicalagin. Punicalin was produced after degradation of punicalagin with tanase (Sigma, 2 U/ml for 8 days), HPLC was used to identify correct punicalagin production; urolithin A and B were from Kylolab S.L. (Murcia, Spain); BAPTA-AM, E-64, pluronic acid, MMP408, MMP9 inhibitor I, GM 6001, recombinant caspase-1, the caspase-1 inhibitor Ac-YVAD-AOM, the caspase-3/7 Inhibitor II Ac-DNLD-CHO, the caspase-3 Inhibitor I Ac-DEVD-CHO, and the specific caspase-1 substrate z-YVAD-AFC were from Merk-Millipore (Darmstadt, Germany); monosodium urate crystals were from Enzo Life Sciences (Farmingdale, NY, USA); A438079 was from Tocris (Ellisville, MO, USA); Yo-Pro-1 iodide, Fura-2 AM, Coelenterazine H, and Cholera Toxin Subunit-Alexa Fluor 647 (CTB-AF647) were from Life Technologies (Paisley, UK); Fuse-It liposomes with 549/565 nm labelled amphipathic molecules within their shell and 480/501 nm labelled marker particles within their lumen were from Ibidi (Martinsried, Germany); the general caspase inhibitor Z-VAD-FMK (zVAD) was from Promega (Madison, WI, USA); Melittin was synthesised by solid phase and was a generous gift of Dr. L Rivas (Centro Nacional Biotecnología, Madrid, Spain); horseradish peroxidase–anti-*β*-actin (C4; sc-47778HRP), rabbit polyclonal antibody to caspase-1 p10 (M-20; sc-514), anti-IL-1*β* (H-153; sc-7884), and anti-ASC ((N-15)-R; sc-22514-R) were from Santa Cruz Biotechnology (Dallas, TX, USA). Mouse monoclonal anti-NLRP3 (Cryo-2; AG-20B-0014) was from AdipoGen (Epalinges, Switzerland). Secondary antibodies for immunoblot analysis (ECL horseradish peroxidase conjugate–linked whole sheep antibody to mouse IgG (NA931V) and ECL horseradish peroxidase conjugate-linked donkey antibody (F(ab′)2 fragment) to rabbit IgG (NA9340V)) were from GE Healthcare (Uppsala, Sweden).

### Membrane binding and pore formation experiments

#### Expression and purification of recombinant protein

Murine IL-1*β* variants (mature and pro form) were expressed as recombinant fusion proteins in *Echerichia coli* strain W3110Z1 (18 °C for 5 h in LB medium) using the expression vectors pQE30 (Qiagen, Hilden, Germany). C-terminal GFP-fusions were expressed in *E. coli* BL21-Codon+ (DE3)-RIL (Agilent Technologies, Boeblingen, Germany) (18 °C for 6 h in LB medium) using pET-15b vectors. Expression of FGF2-Y81pCMF was performed as described before.^9, 16^ All proteins were affinity purified through a N-terminal His-Tag using standard procedures.^9^

#### Preparation of liposomes

All lipids were purchased from Avanti Polar Lipids (Alabaster, AL, USA). Lipid mixtures were first prepared in chloroform that was evaporated under nitrogen stream. The resulting lipid film was further dried and then resuspended to form liposomes with a final lipid concentration of 4 mM (binding experiments) or 8 mM (carboxyfluorescein dequenching experiments).^9, 16^ Unilamellar liposomes were made by 10 freeze/thaw cycles followed by size extrusion through a 400 nm filter for 21 times (Avanti Polar Lipids mini-extruder). Analysis of liposome preparations using dynamic light scattering (Wyatt Technology DynaPro NanoStar, Dernbach, Germany) showed a size distribution of 200–400 nm in diameter.

#### Biochemical analysis of protein binding to liposomes

Liposomes with plasma membrane-like lipid composition containing 2 mol % PI(4,5)P_2_ and phosphatidylcholine liposomes containing 10 mol % PI(4,5)P_2_ were made as previously described.^15, 18^ After incubating mature IL-1*β*, pro-IL-1*β*, and FGF2-Y81pCMF (5 *μ*M), membranes were reisolated by flotation. Material was analysed by SDS-PAGE under reducing conditions (loading about 2.25% load and fractions 1–4) followed by western blotting using anti-IL-1*β* (antibodies-online GmbH, Aachen, Germany) and anti-FGF2 antibodies, respectively. Signals were acquired using an Odyssey infrared imaging system and quantified using Image Studio Software Version 2.1.10 (LI-COR Bioscience, Bad Homburg, Germany).

#### Flow cytometry-based protein binding to liposome analysis

Mature IL-1*β*, pro-IL-1*β*, and FGF2-Y81pCMF (1 *μ*M) were incubated with membranes for 2 h at 25 °C. Protein-bound liposomes were washed by centrifugation (15000 × *g*; 25 °C; 10 min). The liposome pellet was resuspended followed by fluorescence-activated cell sorting (FACS) measurements using a FACS Calibur (Becton Dickinson, Heidelberg, Germany) and data processing using CellQuest Pro software as previously described.^18^

#### Analysis of membrane pore formation

Membrane pore formation by mature IL-1*β*, pro-IL-1*β*, and FGF2-Y81pCMF was analysed as described previously.^9^ Liposomes with plasma membrane-like lipid composition containing 2 mol % PI(4,5)P_2_ and phosphatidylcholine liposomes containing 10 mol % PI(4,5)P_2_ were prepared with 100 *μ*M membrane-impermeant fluorophore 5(6)-carboxyfluorescein (CF, Sigma). For removal of extraluminal CF, first liposomes were harvested by centrifugation at 15 000 × *g* for 10 min at 20 °C, followed by size exclusion chromatography using a PD10 column (GE Healthcare). Afterwards, mature IL-1*β*, pro-IL-1*β*, and FGF2-Y81pCMF (2 *μ*M) were incubated with liposomes and fluorescence dequenching was measured using a SpectraMax GeminiXS fluorescence plate reader (Molecular Devices, Biberach an der Riss, Germany). At the end of each experiment, Triton X-100 (0.2% (w/v) final concentration) addition enabled to assess maximal dequenching that was used to normalise data.

### Cells and treatments

Immortalised BMDMs (iBMDMs) were transduced by lentivirus to stably express IL-1*β* tagged with the fluorescent protein Venus (IL-1*β*Venus). Alternatively, iBMDMs were transiently transfected with IL-1*β*Venus or HEK293 cells with P2X7 receptor and NLRP3 tagged with YFP and Luciferase using Lipofectamine 2000 according to the manufacturer's instructions (Life Technologies). iBMDMs expressing IL-1*β*Venus or ASC-mCherry^34^ were maintained in Dulbecco's modified Eagle's medium (DMEM) supplemented with 10% (heat inactivated) fetal bovine serum (FBS), 1% (2 mM) L-glutamine Q, and 1% penicillin–streptomycin. HEK293 cells were maintained in DMEM/F12 (1 : 1) supplemented with 10% FCS, 2 mM Glutamax, and 1% penicillin–streptomycin. Primary BMDMs were obtained from WT C57BL/6, *Nlrp3*^−/−^,^39^ or *Casp1*^−/−^^40^ and differentiated *in vitro* with 20% of L-cell media as reported elsewhere.^41^ Mouse neutrophils were isolated from bone marrow using the Mouse Neutrophil Enrichment Kit (StemCell Technologies, Vancouver, Canada) according to the manufacturer's instructions. Purity of enriched neutrophils ranged from 73 to 75% as assessed by flow cytometry with double staining for CD11b (MCA74A488, AbD Serotec, Oxford, UK) and Ly-6G (clone 1A8, TonboBiosciences, San Diego, CA, USA). To stimulate the release of IL-1*β*, cells were primed with bacterial endotoxin (LPS, 1 *μ*g/ml for macrophages or 100 ng/ml for neutrophils, 4 h – signal 1) and then treated with ATP (5 mM, from 0.5 to 1 h as stated in figure legends – signal 2) in physiological solution consisting of NaCl (147 mM), HEPES (10 mM), glucose (13 mM), CaCl_2_ (2 mM), MgCl_2_ (1 mM), and KCl (2 mM). Alternatively, after LPS priming cells were stimulated with nigericin (5 *μ*M, 30 min), hypotonic solution (90 mOsm, achieved by diluting the physiological solution 1 : 4 with distilled sterile water), MSU crystals (200 *μ*g/ml, 16 h), or dsDNA delivered using Lipofectamine2000 (1 *μ*g DNA, 16 h).

### Live cell imaging

Cells were plated onto 35 mm-glass bottomed dishes (Greiner Bio-One, Monroe, NC, USA) and incubated on the microscope stage at 37 °C in humidified 5% CO_2_. For monitoring membrane permeabilisation, cells were incubated with PI (5 *μ*g/ml). A Zeiss LSM710 (Oberkochen, Alemania) confocal microscope with a Plan-apochromat × 63 1.3 NA oil immersion and × 40 1.3 NA objectives was used to visualise release of IL-1*β*. Image capture was performed using the ‘Zen 2010b SP1' Zeiss software. Alternatively, macrophages were imaged with a Nikon Eclipse Ti microscope equipped with a 40 × /0.60S Plan Fluor objective and a digital Sight DS-QiMc camera (Nikon, Tokyo, Japan) and the NIS-Elements AR software (Nikon). Time-lapse microscopy images were quantified either with ImageJ (US National Institutes of Health, Bethesda, MD, USA) or Cell Tracker (version 0.6, Pittsburgh, PA, USA).^42^ To quantify changes in plasma membrane dynamics over the time, macrophages were labelled with CTB-AF647 (1 : 1000 dilution) for 30 min at 37 °C and imaged using the Nikon Eclipse Ti microscope as stated above. Inverted fluorescence images converted to grey scale were used for quantification of the mean grey value as relative fluorescence units (RFUs) in different regions of interest of the plasma membrane of each cell using ImageJ (US National Institutes of Health).

### Fluorescence microscopy

BMDMs or immortalised ASC-mCherry macrophages were seeded on coverslips. For phosphatidylserine immunostaining, cells were incubated with Annexin V-FITC (BD Biosciences, Franklin Lakes, NJ, USA) for 10 min at room temperature according to the manufacturer's instructions. To study liposome fusion to the plasma membrane, cells were incubated during 120 min at 37 °C with the Fuse-It liposomes (Ibidi), prepared according to the manufacturer's instructions. Then, cells were fixed with 4% formaldehyde in PBS for 15 min, washed three times with PBS, and images were acquired with a Nikon Eclipse Ti microscope as stated above but using a 60 × Plan Apo Vc objective (numerical aperture, 1.40) and a Z optical spacing of 0.2 *μ*m. Images were deconvolved using ImageJ software (US National Institutes of Health) with Parallel Iterative Deconvolution plugin, and maximum-intensity projections images are shown in the results.

### Fura-2 AM and Yo-Pro uptake assays

Changes in free intracellular calcium concentration were measured with the fluorescent indicator Fura-2 AM. BMDMs were loaded for 40 min at 37 °C with 4 *μ*M Fura-2 AM and 0.02% pluronic acid, washed, and fluorescence was recorded by an automatic fluorescence plate reader (Synergy Mx; BioTek, Winooski, VT, USA) for 200 s at 4 s intervals at a wavelength emission couple 340/380 nm, emission 510 nm. ATP was automatically injected into the wells at the designated time points. Intracellular calcium levels were expressed as the ratio of the emission intensities at 340 and 380 nm, and the value was normalised to the fluorescence at time 0 (F/F_0_). For Yo-Pro uptake, BMDMs were preincubated for 10 min at 37 °C with 25 *μ*M of punicalagin, following the addition of 2.5 *μ*M Yo-Pro. The images were recorded at 10 s intervals for 60 min before and during injection at 37 °C with ATP, digitonin, or Triton-X100. Yo-Pro bound to DNA fluorescence was measured at 485±9/515±9 nm with bottom excitation/emission in the Synergy Mx plate reader (BioTek). In some experiments Yo-Pro uptake was acquired with a Nikon Eclipse Ti microscope as stated above but using a 20 × objective and time-lapse images were analysed with ImageJ software (US National Institutes of Health) and average of relative fluorescence units recorded from ROI are shown in the results.

### Western blotting and ELISA

After cells were stimulated, cell supernatants were collected and centrifuged at 300 × *g* for 8 min at 4 °C to remove detached cells and generate a cell-free preparation. Before western blot analysis, proteins in supernatants were concentrated by centrifugation at 11 200 × *g* for 30 min at 4 °C through a column with a cut-off of 10 kDa (Microcon; Merk-Millipore). Detailed methods used for cell lysis and western blot analysis had been described previously.^43^ The ELISA kits for mouse IL-1*β*, IL-6, TNF-*α*, and IL-1*α* were from R&D (Minneapolis, MN, USA) and were used following the manufacturer's instructions.

### Bioluminescence resonance energy transfer measurements

Transfected HEK293 cells were plated on a poly-L-lysine-coated 96-well plate; after adhesion, cells were washed with PBS with calcium and magnesium, and readings were performed immediately after the addition of 5 mM coelenterazine-H substrate in physiological solution. Signals were detected with two filter settings (Renilla-luciferase (Luc) filter (485±20 nm) and YFP filter (528±20 nm)) at 37 °C using the Synergy Mx plate reader before and after automatic ATP injection. The bioluminescence resonance energy transfer (BRET) ratio was defined as the difference between the emission at 530 nm/485 nm of R-Luc and YFP NLRP3 fusion protein and the emission at 530 nm/485 nm of the R-Luc fusion NLRP3 alone. Results are expressed in milliBRET units normalised to basal signal.

### LDH release, caspase-1 activity, and K^+^ measurements

The presence of LDH in cell supernatants was measured using the Cytotoxicity Detection kit (Roche, Mannheim, Germany), following the manufacturer's instructions. It was expressed as the percentage of the total amount of LDH in the cells. In some control experiments, 25 *μ*M of punicalagin was mixed with a lysate of BMDMs before LDH activity measurement. Caspase-1 activity was measured monitoring the cleavage of the fluorescent substrate z-YVAD-AFC at 400/505 using a Synergy Mx plate reader (BioTek) during 30 min intervals during 6 h. Results are presented as the slope of the RFUs/min. Intracellular K^+^ was quantified from BMDM cell lysates by indirect potentiometry on a Cobas 6000 with ISE module (Roche).

### Statistical analysis

Data are presented as mean±S.E.M. from the number of assays indicated (from at least three separate experiments). Data were analysed, using Prism (GraphPad, La Jolla, CA, USA) software, by an unpaired two-tailed Student's *t-*test to determine the difference between two groups or by one-way ANOVA with the Bonferroni multiple comparison test to determine the differences among more than two groups.

## Abbreviations

AIM2: absent in melanoma 2
ASC: apoptosis-associated speck-like protein containing a caspase recruitment domain
BRET: bioluminescence resonance energy transfer
DAMP: damage-associated molecular pattern
FGF2: fibroblast growth factor 2
IL-1*β*: interleukin-1*β*
LDH: lactate dehydrogenase
LPS: lipopolysaccharide
NLR: Nucleotide-binding domain and Leucine-rich repeat containing Receptor
NLRP3: NACHT, LRR, and PYD domains containing protein 3
PAMP: pathogen-associated molecular pattern
PRR: pattern recognition receptor
PI: propidium iodide
WT: wild type
