# Supplementary figures and images for: Inflammasome-dependent IL-1β release depends upon membrane permeabilisation
Source: Cell Death Differ. 2016 Feb 12;23(7):1219–31. doi: 10.1038/cdd.2015.176 (PMC4946890; doi:10.1038/cdd.2015.176)

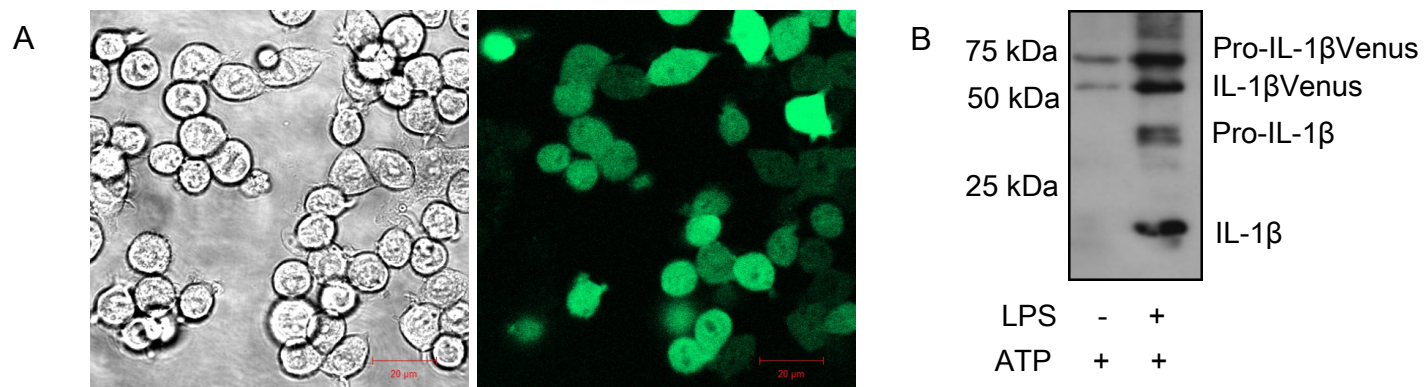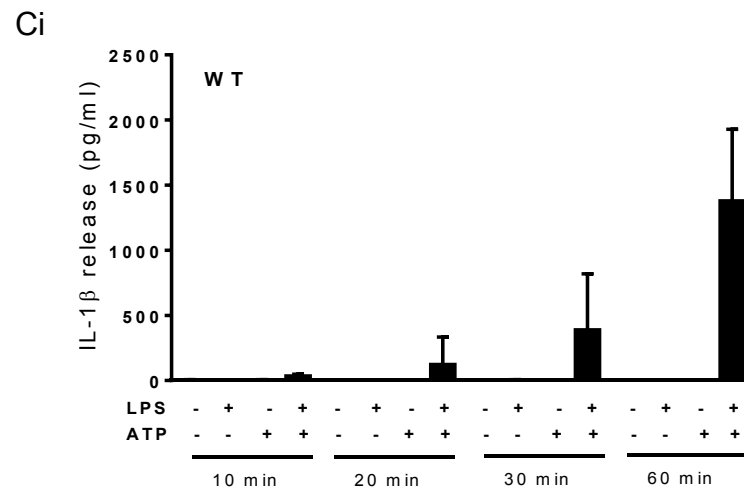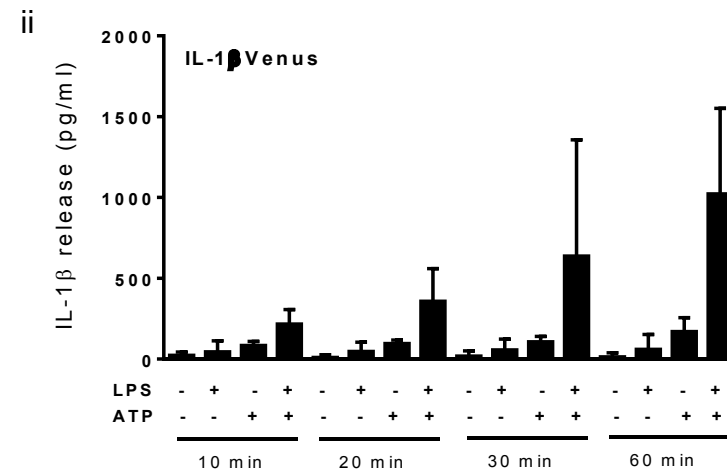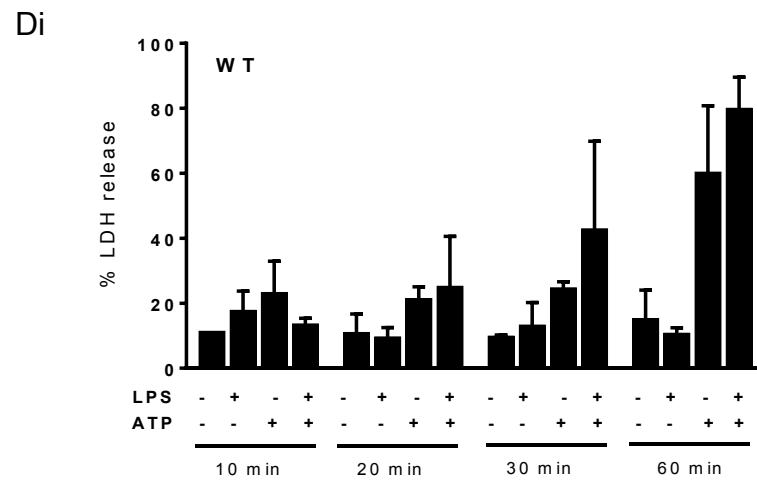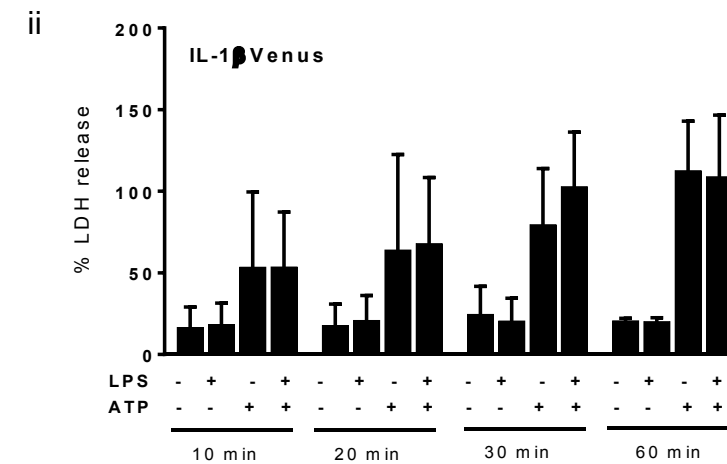

Supplement: Supplementary Figure 1 [file cdd2015176x1.pdf]

# A

Punicalagin (MW 1084 g/mol)

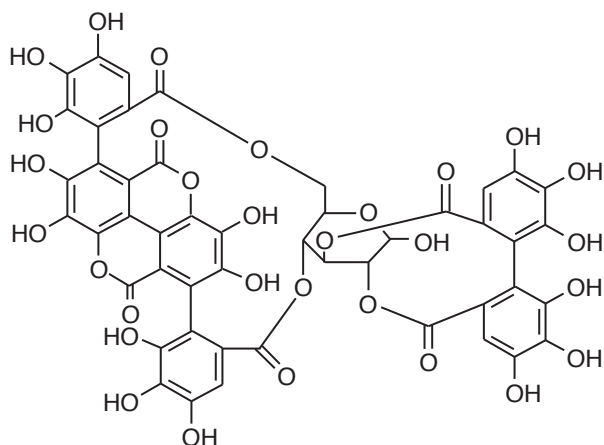

Punicalin (MW 782 g/mol)

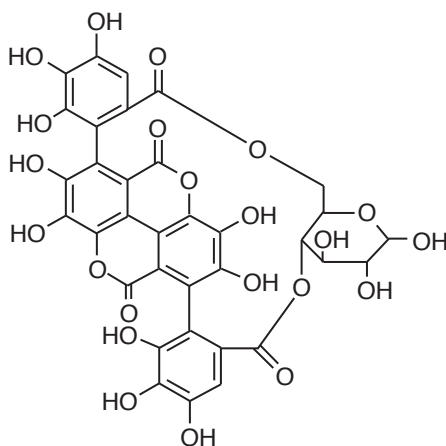

Ellagic acid (MW 302 g/mol)

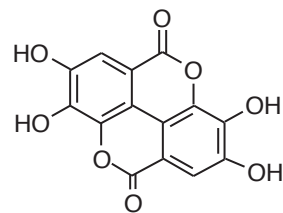

Urolithin A (MW 228 g/mol)

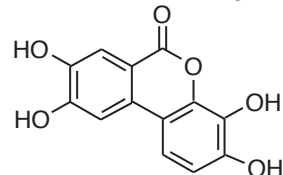

# B

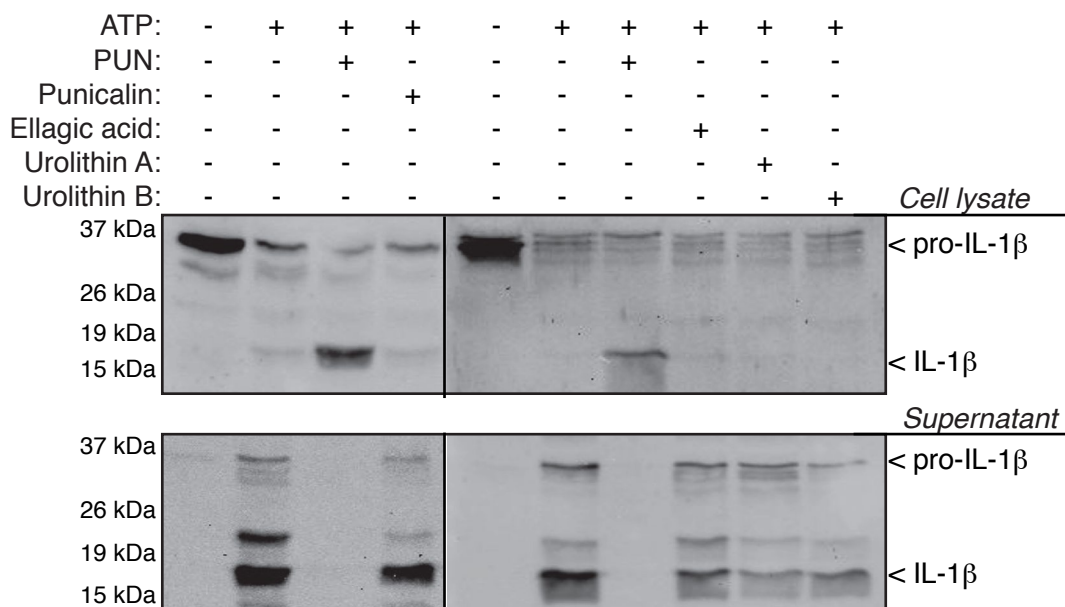

# C

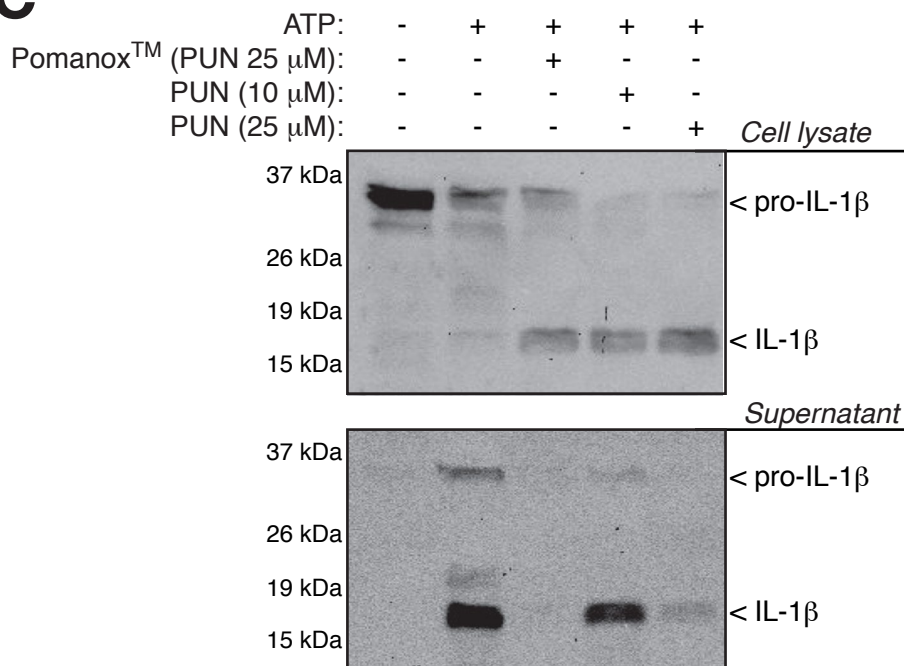

Supplement: Supplementary Figure 2 [file cdd2015176x2.pdf]

**A**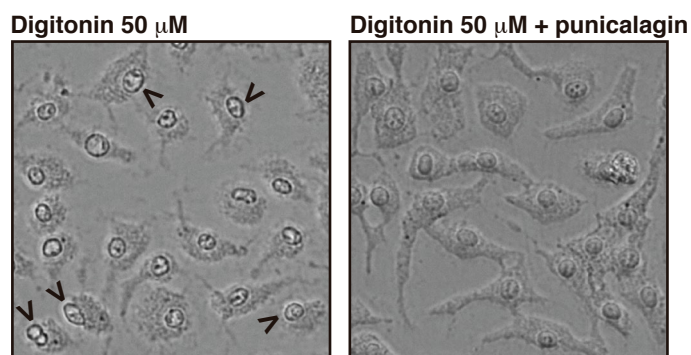**B**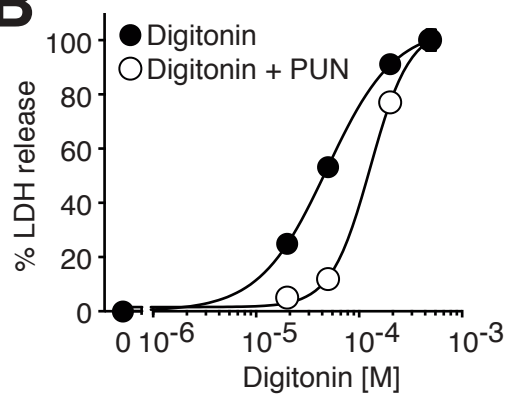**C**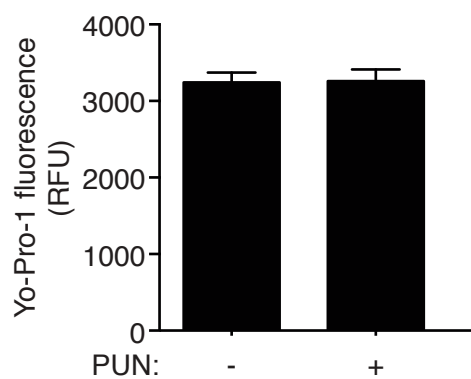**D**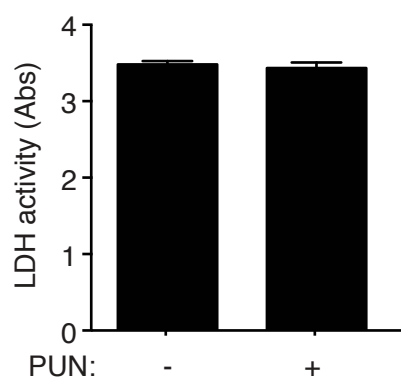**E**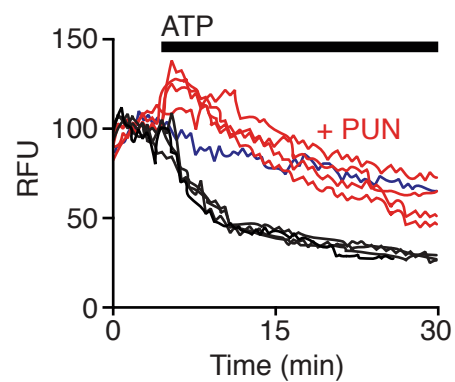

Supplement: Supplementary Figure 3 [file cdd2015176x3.pdf]

**A**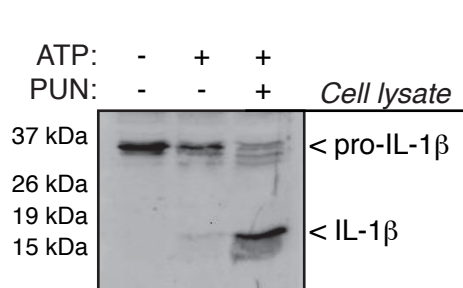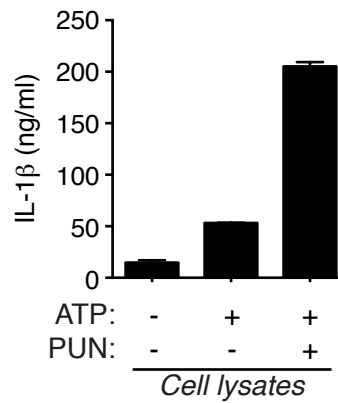**B**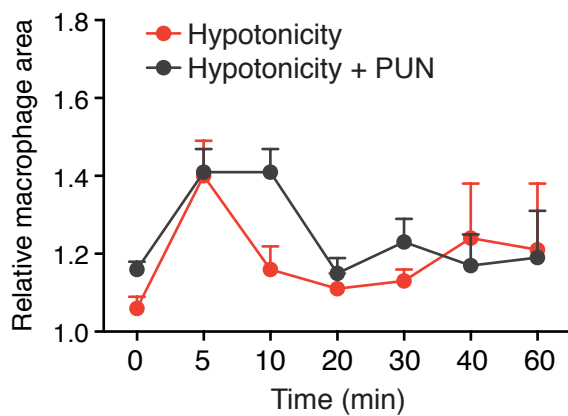**C**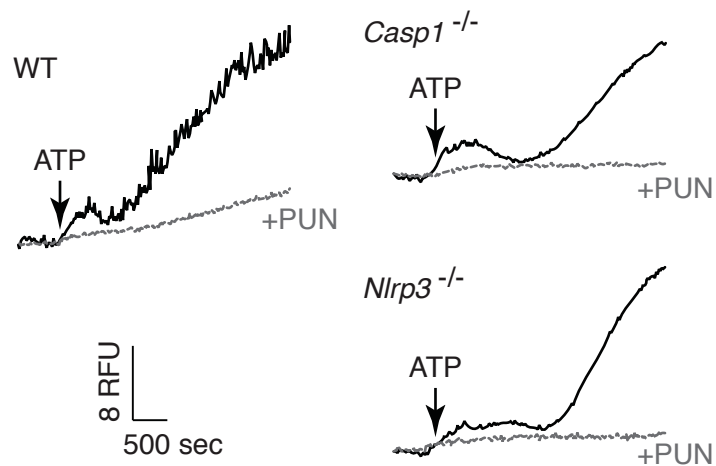**D**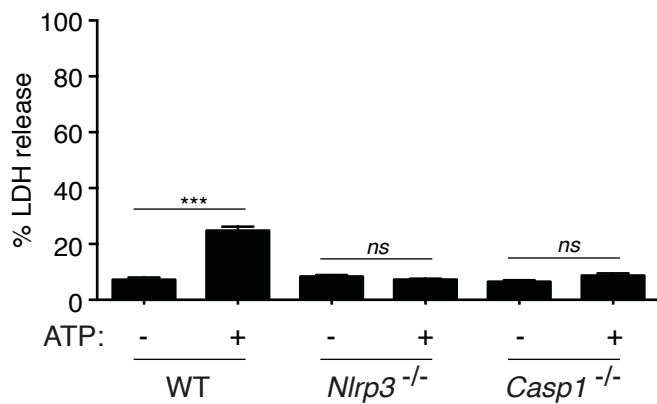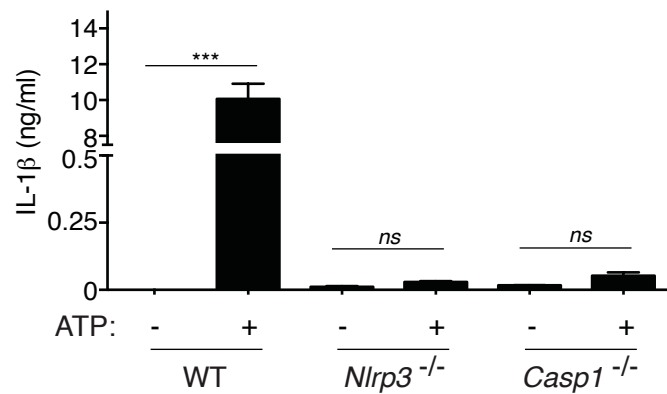**E**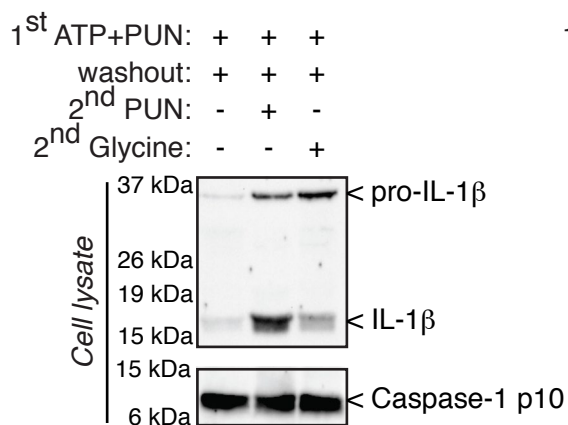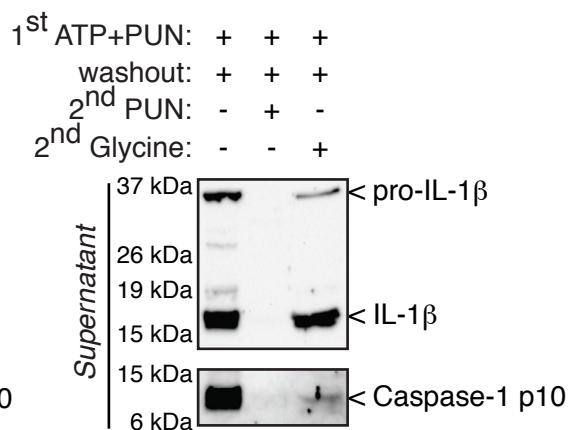

Supplement: Supplementary Figure 4 [file cdd2015176x4.pdf]
