# Supplementary material for: Inflammasome-dependent IL-1β release depends upon membrane permeabilisation
Source: Cell Death Differ. 2016 Feb 12;23(7):1219–31. doi: 10.1038/cdd.2015.176 (PMC4946890; doi:10.1038/cdd.2015.176)
Supplement: Supplementary Legends [file cdd2015176x14.doc]

**Supplementary Figure Legends**

**Supplementary Figure 1:** *Establishment of IL-1βVenus expressing cells.* Immortalised BMDMs were transduced by lentivirus to express pro-IL-1βVenus. Shown is a bright-field image of the BMDMs in culture and the corresponding fluorescence image showing the sub-cellular distribution of the pro-IL-1βVenus (A). Scale bar represents 20μm. These BMDM-IL-1Venus cells were treated plus or minus with LPS (1μg/ml, 4h) and then with the ATP (5mM, 1h). Supernatants were collected and blotted for IL-1β. As seen in panel (B) secretion of tagged or endogenous IL-1β requires LPS priming. The band at 58kDa is pro-IL-1βVenus, at 44kDa is IL-1βVenus, at 31kDa is endogenous pro-IL-1β, and at 17kDa is endogenous IL-1β. These data confirm that the transduced cells are competent for inflammasome activation. WT (i) or IL-1βVenus expressing cells (ii) were treated with LPS (1μg/ml, 4h) and then ATP (5mM) for 10, 20, 30 or 60min with supernatants analysed for IL-1β (C) and LDH (D). Data are the Mean ± SEM, ELISA n=4, LDH n=3.

**Supplementary Figure 2:** *Punicalagin structure necessary to prevent IL-1β release.* (A) Structure of punicalagin molecule and their component parts punicalin, ellagic acid and urolithin. (B) Immunoblot analysis of the processing of pro-IL-1β in cell lysate and supernatant of BMDMs primed with LPS (1 μg/ml, 4h) and unstimulated (-) or stimulated (+) with ATP (5 mM, 30 min) in absence (-) or presence (+) of punicalagin (PUN; 25 µM), Punicalin (25 µM), Ellagic acid (100 µM), Urolithin A (100 µM) or Urolithin B (100 µM). (C) Immunoblot analysis of IL-1β processing in BMDMs treated as in A but in absence (-) or presence (+) of Pomanox^TM^ as a source of punicalagin at 25 µM.

**Supplementary Figure 3:** *Punicalagin maintain cell integrity after detergent treatment.* (A) Representative images of BMDMs primed with LPS (1 µg/ml, 4h) and then treated with digitonin (50 μM, 30 min) in absence or presence or punicalagin (25 µM). (B) Percentage of extracellular LDH release in supernatant from BMDMs treated as in A but with increasing doses of digitonin. (C) Yo-Pro-1 fluorescence on BMDMs primed with LPS (1 µg/ml, 4h) and then treated with ATP (5 mM, 30 min) to obtain maximum fluorescence. Then cells were incubated or not with punicalagin (PUN; 25 µM), and fluorescence signal was excited and recorded from the bottom of the plate. (D) LDH activity measured in BMDM cell lysates incubated or not with punicalagin (PUN; 25 µM), punicalagin did not interfere with LDH enzymatic assay. (E) IL-1βVenus fluorescence quantification in iBMDMs expressing pro-IL-1βVenus primed with LPS (1 µg/ml, 4h) and then untreated (blue trace) or treated with ATP (5 mM, 25 min) in absence (black traces) or presence or punicalagin (25 µM; +PUN, red traces). Each trace represents one independent cell recorded in different days. The cells measured correspond with the cells shown in Supplementary movies 4 and 5.

**Supplementary Figure 4**: *ELISA validation for mature IL-1β*. (A) Immunoblot analysis (left) and ELISA (right) of the same cell lysate samples from BMDMs primed with LPS (1 μg/ml, 4h) and unstimulated (-) or stimulated (+) with ATP (5 mM, 30 min) in absence (-) or presence (+) of punicalagin (PUN; 25 µM). (B) Volume changes of THP-1 macrophages treated with a hypotonic solution (90 mOsm) in the absence or presence of punicalagin (PUN; 25 µM). (C,D) Kinetic of Yo-Pro uptake (C) and percentage of extracellular LDH and ELISA of IL-1β (D) released from wild-type (WT), *Casp1^-/-^Casp11^-/-^* and *Nlrp3^-/-^* BMDMs primed with LPS (1 μg/ml, 4h) followed stimulation with ATP (1 mM, added when indicated with an arrow in C) in the absence or presence of punicalagin (PUN; 25 μM). (E) Immunoblot analysis for IL-1β and p10 fragment of active caspase-1 of cell lysate and supernatant of mouse BMDMs primed with LPS (1 μg/ml, 4h), followed by no stimulation (-) or stimulation (+) with ATP (5 mM, 20 min) and punicalagin (PUN; 25 μM), then the cells were washed and incubated for further 20 min in the absence or presence of PUN or glycine (5 mM). ***p < 0.001; ns, not significant (p>0.05) difference (Student’s *t*-test).

**Supplementary movie 1**: Immortalised mouse BMDMs expressing IL-1βVenus were treated with LPS (1μg/ml, 4h) and imaged using a Zeiss LSM710 confocal microscope. Fluorescence of IL-1βVenus (green) and PI (red) was then observed when were treated with or without ATP (5mM). Image capture was performed using the "Zen 2010b SP1" Zeiss software. The panels in (A) show images of brightfield (upper right quad), Venus (upper left quad), PI (lower left quad) and merged (lower right quad).

**Supplementary movie 2**: Immortalised mouse BMDMs expressing IL-1βVenus were treated with LPS (1μg/ml, 4h) and imaged using a Zeiss LSM710 confocal microscope. Fluorescence of IL-1βVenus (green) and PI (red) was then observed when were treated with or without ATP (5mM). Image capture was performed using the "Zen 2010b SP1" Zeiss software. The panels in (A) show images of brightfield (upper right quad), Venus (upper left quad), PI (lower left quad) and merged (lower right quad).

**Supplementary movie 3**: Immortalised mouse BMDMs expressing IL-1βVenus were treated with LPS (1μg/ml, 4h) and imaged using a Zeiss LSM710 confocal microscope. Fluorescence of IL-1βVenus (green) and PI (red) was then observed. Image capture was performed using the "Zen 2010b SP1" Zeiss software. The panels in (A) show images of brightfield (upper right quad), Venus (upper left quad), PI (lower left quad) and merged (lower right quad).

**Supplementary movie 4**: Time-lapse fluorescence microscopy of iBMDMs expressing pro-IL-1βVenus primed with LPS (1 µg/ml, 4 h) and stimulated with ATP (5 mM). Frames are recorded every 30 sec during 30 min. Monochromatic deconvolved images are shown. Time (min) is presented as a counter inside the movie. Addition of different treatments is also indicated in the movie. Scale bar represent 10 µm.

**Supplementary movie 5**: Time-lapse fluorescence microscopy of iBMDMs expressing pro-IL-1βVenus primed with LPS (1 µg/ml, 4 h) and incubated with punicalagin (25 µM) 20 min before and during ATP (5 mM) stimulation. Frames are recorded every 30 sec during 30 min. Monochromatic deconvolved images are shown. Time (min) is presented as a counter inside the movie. Addition of different treatments is also indicated in the movie (punicalagin addition is marked as PB02). Scale bar represent 10 µm.

**Supplementary movie 6**: Time-lapse fluorescence microscopy of BMDMs primed with LPS (1 µg/ml, 4 h), labelled with cholera toxin B-Alexa fluor 647 and then recorded every 30 sec during 60 min. Monochromatic inverted images are shown. Time (min) is presented as a counter inside the movie. Scale bar represent 10 µm.

**Supplementary movie 7**: Time-lapse fluorescence microscopy of BMDMs primed with LPS (1 µg/ml, 4 h), labelled with cholera toxin B-Alexa fluor 647 and then recorded every 30 sec during 60 min. Punicalagin (PB02; 25 µM) was added after 7 min of recording and is indicated in the movie as PB02. Monochromatic inverted images are shown. Time (min) is presented as a counter inside the movie. Scale bar represent 10 µm.

**Supplementary movie 8**: Time-lapse fluorescence microscopy of BMDMs primed with LPS (1 µg/ml, 4 h), labelled with cholera toxin B-Alexa fluor 647 and then recorded every 30 sec during 35 min. ATP (5 mM) was added after 12 min of recording and is indicated in the movie. Monochromatic inverted images are shown. Time (min) is presented as a counter inside the movie. Scale bar represent 10 µm.

**Supplementary movie 9**: Time-lapse fluorescence microscopy of BMDMs primed with LPS (1 µg/ml, 4 h), labelled with cholera toxin B-Alexa fluor 647 and then recorded every 30 sec during 35 min. Punicalagin (25 µM; marked as PB02 in the movie) was added after 7 min of recording and ATP (5 mM) after 12 min and both events are indicated in the movie. Monochromatic inverted images are shown. Time (min) is presented as a counter inside the movie. Scale bar represent 10 µm.
